# Functional architecture of the forebrain cholinergic system in rodents

**DOI:** 10.21203/rs.3.rs-4504727/v1

**Published:** 2024-06-19

**Authors:** Laszlo Zaborszky, Peter Varsanyi, Kevin Alloway, Candice Chavez, Matthew Gielow, Peter Gombkoto, Hideki Kondo, Zoltan Nadasdy

**Affiliations:** Rutgers University; Rutgers University; Penn State University; Rutgers University; Rutgers, the State University of New Jersey; Swiss Federal Institute of Technology in Zurich (ETH Zurich); Rutgers University; University of Texas at Austin

## Abstract

The basal forebrain cholinergic system (BFCS) participates in functions that are global across the brain, such as sleep-wake cycles, but also participates in capacities that are more behaviorally and anatomically specific, including sensory perception. To better understand the underlying organization principles of the BFCS, more and higher quality anatomical data and analysis is needed. Here, we created a “virtual Basal Forebrain”, combining data from numerous rats with cortical retrograde tracer injections into a common 3D reference coordinate space and developed a “spatial density correlation” methodology to analyze patterns in BFCS cortical projection targets, revealing that the BFCS is organized into three principal networks: somatosensory-motor, auditory, and visual. Within each network, clusters of cholinergic cells with increasing complexity innervate cortical targets. These networks represent hierarchically organized building blocks that may enable the BFCS to coordinate spatially selective signaling, including parallel modulation of multiple functionally interconnected yet diverse groups of cortical areas.

## Introduction

The basal forebrain (BF) corticopetal projection system is the main source of acetylcholine (ACh) for all cortical areas, the hippocampus, and the amygdaloid body. Lesions or blockade of ACh in the cortex results in impairments in perception^1^, cognitive flexibility^2^, executive function, and cortical plasticity^3–5^. During aging, in Alzheimer’s Disease (AD) and other neurodegenerative diseases, the cholinergic space (e.g., the region containing cholinergic projection neurons) shows volume reduction, which correlates with atrophy of their cortical targets and the decline in cognitive functions^6–8^.

Evidence from tracing studies and lesions has suggested that the cholinergic projection system is organized topographically. For example, lesions in posterior BF produce more damage in the cholinergic innervation of the auditory cortex than lesions in more anterior BF locations^9^. However, initially the projections of the BFCS were considered part of a “diffuse cortical projection system”^10^. Several factors contributed to this assumption, including the variable spatial distribution of cholinergic cells across the rostro-caudal extent of the BF, the observation that adjacent BF cholinergic (BFC) cells projecting to distant regions of the cortex appear to have overlapping dendritic fields^11^, and that measurements of cortical ACh release using *in vivo* microdialysis techniques failed to indicate differences between cholinergic activities in different cortical regions during various behaviors^12^. Additionally, certain functional data have contributed to the view that cholinergic signaling in the cortex is slow and non-specific, and likely acts through volume transmission^13–15^.

Recent anatomical studies paint a more complex picture wherein the cholinergic projection to the neocortex is not diffuse but instead is organized into cortical target-specific groups of cholinergic neurons that receive specific combinations of inputs^16–18^. Moreover, cholinergic cells (Ch) that project to the superficial or deep layers of the medial prefrontal (mPFC) and somatosensory cortex in mice are largely separated in the BF^18,19^. Also, new evidence from real-time amperometric recordings indicates cholinergic signaling in attentional contexts is rapid, phasic, transient, and probably synaptic^20^.

In a limited set of rat brains (n = 9), using distinct retrograde tracers deposited into disparate cortical areas to map labeled cells in the BF and cortex, we demonstrated that the BF has a complex topographic organization consisting of segregated or overlapping pools of projection neurons. The overlap in the BF of projecting populations appears to be related to the degree of connectivity between their cortical targets^16^. This complex efferent organization pattern and the specific input pattern to target-identified cholinergic neurons^17^, together with the regionally selective dendritic orientation of cholinergic neurons^21^, led to the hypothesis that the anatomical organization of the BF constitute a “scaffolding” that may enable the cholinergic system to modulate topographically organized interconnected cortical areas^16^. Indeed, optogenetic activation of cholinergic subgroups in the BF induces modality-selective desynchronization in specific sensory cortical areas in transgenic mice^22^, indicating modularity in the organization. Although Buzsaki and colleagues demonstrated decades ago that circumscribed ibotenic acid lesions in the BF result in a dramatic increase of slow delta waves corresponding to the disappearance of cortical AChE fibers^23^, it has never been resolved whether cholinergic neurons modulate disparate cortical regions in a globally or a spatially selective fashion, due to the absence of a viable anatomical model to serve as a basis for generating hypotheses about functional relationships.

Here, as an analytical tool to quantify the organization of the BF we created a “virtual BF” database (http://zaborszkylab.org/3DCholinergicSpace/) incorporating the locations of cholinergic cell bodies quantified in over 70 experimental brains with cortical tracer injections. Some of the brains were used in previous publications^9,16,17,24^. All brains are identified with a specific ID and listed in Table S1 with their source. We used this virtual BF to further examine whether the spatial location of cells projecting to 30 ontologically defined cortical targets show systematic organizational features that deviate from the random distribution suggested by the diffuse model. We quantified the local co-occurrence of functionally related cholinergic neurons by computing the correlation between the local density (in the BF) of those neurons classified based on their cortical projection targets. We devised various algorithms collectively referred to as ‘spatial density correlation’ (SDC) to capture the efferent organizational principles of the BFCS, facilitating the understanding of the underlying mechanisms of its complex functions.

## Results

### Data production and collections

Cholinergic neurons in the BF were mapped with the Neurolucida^R^ system in 73 rat brains that received pairwise conventional retrograde (Fast Blue [FB] and Fluoro-Gold [FG]) or virus tracer injections in the cortex (n = 106). After mapping fluorescently labeled cells from 200 μm series, sections were re-stained with Nissl, and images of sections were aligned to a reference brain with 50 μm series. We implemented a relational database schema within the PostgreSQL framework that communicates with the QGIS mapping client to store and visualize 3D vectorial cell data, including delineations and images. This solution provided a complete set of functions for analyzing cell densities, determining spatial relationships, and manipulating delineations with very fast and smooth visualization of high-resolution images as well as vectorial data. We created a reference brain from a full series of 50 μm sections of a single brain in which all cholinergic cells were mapped irrespective of their targets, and then sections were stained for Nissl. These reference sections were imaged with 3 μm/pixel resolution and aligned in 3D. The software (Java, ImageJ) was able to slice this high-resolution 3D stack of images in any plane to match sections from the experimental brains. The spatial registration of experimental sections to the reference brain is carried out using mixed rigid body, affine, and B-Spline-based elastic transformations (Extended Data Fig. 1).

All injection sites were warped into specific Paxinos atlas plates and collected in a PDF file (Extended Data Fig. 2). Extended Data Fig. 3 validates the preciosity of warping. These maps are important in the normalization procedures applied in the density analysis (see METHODS). Intraoperative electrophysiology was used to identify various motor^25^, somatic sensory^25^, auditory^9^, and visual cortical areas (Gielow et al., 2013, SFN Abstract). Table S1 details the percentage participation of each injection in the Paxinos cortical areas, appended with notes about the electrophysiological identification of modality-specific cortical areas. Table S2 explains the cortical ontology categories used in our study.

### Spatial density correlations of cholinergic neurons in the BF

Projection Ch neurons from all experimental brains were co-registered to a reference brain. Next, we defined a 300 μm radius spherical volume around each of the 5,674 projection neurons and counted the number of cells projecting from this volume to any of the 30 cortical ontologically defined regions. This provided us with a 5,674 × 30 matrix of projection counts. In this matrix, each cortical area (column) was associated with a vector of 5,674 rows representing all the BFC neurons projecting to that cortical area, hereafter referred to as target-projection vector (TPV). Hence, if two cortical areas receive input from the same group of BFC neurons and those neurons display similar cortical projection patterns, their column-vectors are expected to be highly correlated. Conversely, if two cortical areas receive input from different groups of BFC neurons, their column vectors are expected to be uncorrelated. Figure 1 displays the methods used for spatial density correlations and Fig. 2 represents the correlation matrix of pairwise correlations between each of the 30 cortical areas. The numbers in the matrix represent the coefficients of Pearson correlations between all pairs of TPVs, quantifying the similarity of cortical projection patterns within a local cholinergic ensemble. The symmetrical red and blue areas represent the most and least likely composition of cortical projection neurons within 300 μm ensembles identified by their cortical projections in the BF, respectively.

The order of cortical areas in Fig. 2 is arbitrary, and this fragments the high-correlation areas unless we reorganize them by placing similar correlation values next to each other. To achieve this and unravel the underlying architecture, we computed the linkage, by subjecting the matrix on Fig. 2 to a hierarchical clustering using the ‘*complete linkage*’ procedure. We ordered rows or columns based on their minimum squared different sums (Euclidean distance), which resulted in a scalar value. For example, the algorithm merges the rows ‘M1-forelimb’ (M1FL) and ‘S1-forelimb’ (S1FL), and these values are compared with the next most similar row, which is the ‘S1-orofacial’ cluster, and so on (Fig. 3). In other words, this hierarchically reorganized matrix represents the classification of the BF cell populations according to their neighboring cellular compositions with respect to their cortical targets. Hierarchical clustering revealed three well-articulated clusters of BFC neuron populations classified as: (i) Somatic Sensory-Motor (SSM), (ii) Visual, (iii) and Auditory branches, as defined by the sensory modality most predominant within them. This is of particular significance considering that we did not impose any order of structural categories via the experimental design when selecting the injection sites. In Fig. 2, we list the cortical areas in alphabetical order, while Fig. 3 shows the order after applying hierarchical clustering on the data. This method was agnostic to the structural and topographical relationship between the areas -- the three networks emerged exclusively from the correlation between projection patterns expressed by the BFC neurons. Figure 4B shows the three large clusters in a 3D rendering. Please note that the orbitofrontal cortex (OFC: VO, LO) and the medial PFC (mPFC: PrL, IL) are split into two networks; OFC in the SSM, while mPFC, and visual cortex are together with the hippocampus, while the amygdala is classified with the auditory cortex.

The statistical validation of the resulting dendrogram verified the three large clusters. We calculated the *p*-values for the dendrogram via multiscale bootstrap resampling^26,27^. Extended Data Fig. 4. shows that the large clusters are well within the 90% confidence level. In addition, to hierarchical clustering, we applied principal component analysis to the correlation matrix and found that the two largest eigenvalues, representing 37% and 43.8% of the variance, were associated with the exact same SSM, Visual, and Auditory networks (Fig. 5). We conclude that the target composition of the local cholinergic ensembles regarding their cortical projections is far from the random uniform distribution. Instead, they connect functionally related cortical targets with high precision.

The next question was whether the network underlying the axonal projections to functionally related cortical targets is topographically clustered or homogeneously distributed in the BFC. In other words, do three functional network projections originate from anatomically restricted BF areas or are they homogeneously distributed? The hierarchical clustering result could afford both. To test this, we selected high-density cell populations among the highest correlating intersections. For instance, Fig. 6 illustrates the co-distribution spaces along the V1 ‘bundle’ of Ch cells. While Cg1 (cingulate) projecting Ch cells share space with V1 rostrally, PrL (prelimbic) innervating Ch cells share space with V1 Ch cells in a small aggregate, both rostrally and caudally. Finally, RS (retrosplenial) projecting Ch cells share space with V1 Ch cells in a larger wedge-shaped space between the rostral and caudal V1/PrL group. Notably, these cells represented in Fig. 6 are not the only cell populations present in the specified spaces; there are other cells targeting different cortical areas with less density and lower correlation.

The location of the normalized cell populations projecting to individual cortical regions used in the hierarchical clustering were plotted in 2D sections of the 3D template brain, arranged at 11 consecutive levels encompassing the whole extent of BF. The rows in Fig. 7 represent these sections along the antero-posterior axis. To compare the local distribution of normalized projection populations with the hierarchical cluster matrix, the rows are organized in the same order as the dendrogram in Fig. 3. Each row shows the leaf of the dendrogram representing the hierarchical organization of the BFC cell populations. As seen in Fig. 7, the distribution of cell populations shows a systematic transition along the antero-posterior axis of the BF while also revealing the three main functional groups labeled after its predominant sensory modality as Auditory, SSM, and Visual, as evident from Fig. 4. In other words, the clusters of highly correlated projections in the matrix of Fig. 3 correspond to specific locations in BF.

### Somatic Sensory-Motor (SSM) network

According to the dendrogram of Fig. 4A, the smallest Euclidean distances as low-level similarity environment is represented by *cluster 4*, between motor forelimb (M1FL) / sensory forelimb (S1FL); and by *cluster 13* between motor hindlimb (M1HL) and S2 cortex. It must be kept in mind that even at this level of similarity, these clusters don’t represent independent spaces but show **overlap in unique** combinations. At the next similarity level S1 orofacial joins to *cluster 4 and 10* constructing *cluster 16* and cluster 16 joins with cluster 13 creating cluster 21. One level higher, *clusters 23 and 25* create *cluster 27*. In *cluster 27*, all clusters representing the somatic-sensory motor network are merged. The cophenetic distance, a scalar value that is calculated from the Euclidian distance, is the measure of intergroup dissimilarity at which two clusters first combine into a single cluster. In Extended Data Fig. 4, a scale at the left helps to estimate the cophenetic distance, and in Table S3 the numerical values for Euclidian and cophenetic distances for all target combinations can be looked up. For the 29 cluster combinations these two numerical values are the same (Sheet 2, Table S3). As can be surmised, the SSM network is composed of two subnetworks.

### Visual network

V1 and V2 visual areas were defined, following craniotomy and durotomy, by micropipette electrodes inserted into the right cerebral cortex to detect visually evoked potentials in response to strobe light stimulation of the contralateral eye. Visually responsive areas were marked, and a separate pipette was inserted into the same cortical area at two or more such responsive locations, and the retrograde tracer Fast Blue was deposited iontophoretically at a depth of up to 1.3 mm ventral to pia (Gielow et al., 2013, SFN Abstract). In addition to V1 and V2-projecting cholinergic cells, this network also contains cells projecting to the retrosplenial (RS), Cg1, PrL, and IL (infralimbic) cortex. Medial (MEC), lateral entorhinal cortex (LEC), and hippocampal-projecting cholinergic cells cluster to this network using the Euclidian similarity analysis. Figure 6 shows the 3D rendering of Ch cells to V1, Cg1, RS, and PrL, viewed at 6 coronal levels.

### Auditory network

Primary (A1) and secondary (A2) auditory fields were identified with multi-unit extracellular recordings as described^9^. A1 was defined by a general progression of low to high characteristic frequency (CF) along the posterior to anterior axis. Anterior placements that resulted in a reversal in the CF progression were defined as lying in the anterior auditory field (AAF^28^). Furthermore, the posterior dorsal auditory field (PDAF) belt area was identified as more responsive to noise than to tone^28^. We also identified the suprarhinal auditory field (SRAF^29,30^), as the auditory-responsive region just above the rhinal sulcus. CF was defined as the frequency that elicited an evoked discharge at the lowest dB sound pressure level (SPL), if more than one stimulus frequency was elicited at the same dB SPL, then the frequency that elicited the greatest number of spikes was identified as the CF. Auditory fields AAF, PDAF, and SRAF are also termed A2 fields.

In addition to auditory cortical-projecting Ch cells, this network, via similarity measurements, contains cholinergic clusters projecting to perirhinal (PER), postrhinal (POR), insular cortex (AIP, IDIGI) and the amygdala. The shortest similarity distance has been found in this network between A1 and SRAF, between PDAF and AAF, between AIP and POR, and between IDIGI (insular cortex, dysgranular and granular parts) and the Amygdala (Extended Data Fig. 4). The anterior belt region (AAF) overlaps with various somatosensory fields, including barrel fields (D-row) and upper lip region, but belongs to the SSM network based on the Euclidian and cophenetic distances.

### Relation of distance of cortical injections to average BF cell distance

Using the 3D distances between the injection site centroids and the average distance of each cell pair gives a correlation of 0.249; separately calculating correlations for the three large networks: in the auditory network this correlation is 0.569, in the SSM network 0.347, and 0.065 in the visual network. We then computed the correlation between the row vector distance of the correlation matrix and the median cell distance between projection cell populations for each combination of cortical areas (420 combinations: [30 × 30/2]-30), resulting in a correlation coefficient of 0.629 (Extended Data Fig. 5).

### Double labeled cells

Double-labeled (DL) cholinergic neurons are relatively few in numbers. Of 52 cases out of the 73 brains, only 20 brains contained one or more DL cells in our database (Table S1). In a collection of new additional cases out of 20 brains 9 brains contained one or 2 DL cells; 7 brains 5–12 such cells; in additional four brains no DL cells were found in 200 μm series (Kondo and Zaborszky, in preparation).

## Discussion

We found a precise relationship between the cellular composition of local BFC ensembles and their cortical targets by analyzing the projection pattern of 5,674 cholinergic neurons in the BF labeled by their cortical targets. Accordingly, local BFC ensembles (~ 300 μm radius) target functionally related cortical areas. In other words, the cellular composition of local BFC networks reflects the hierarchical organization of cortical functions. This may suggest that these BFC nodes can dynamically coordinate the activity of diverse but functionally connected cortical areas.

Two hypotheses motivated our methodology. According to the first one, the BFC system is organized in locally networked cellular ensembles, as opposed to the formation of a diffuse network^10,31^. These local ensembles project in specific combinations to cortical targets, and different ensembles project to different combinations of cortical targets. According to the second hypothesis, the combination of cortical targets in each BFC ensemble is also far from random; instead, it is somewhat organized. Their projection pattern may follow the topography of cortical areas, i.e., nearby Ch cells project to topographically nearby cortical areas or functionally related but not necessarily adjacent areas. We developed and applied a Spatial Density Correlation (SDC) method to define the target composition of local BFC neurons within 300 μm cell-centered volumes. This revealed the diversity of these BFC ensembles regarding their cortical projection patterns (Fig. 3). While the association between some cortical targets and local BFC ensembles was strong (r > 0.7), those highly correlated fields diffusely scattered over the arbitrarily ordered list of cortical areas. The underlying architecture did not emerge until we subjected the matrix of SDC to hierarchical clustering. The application of hierarchical clustering on cholinergic cell density normalized according to 30 ontologically defined cortical regions resulted in a robust hierarchy, organized into three well-defined clusters aligned with (i) auditory, (ii) visual and (iii) somatosensory/motor functional groups. This hidden organization pattern challenges the alternative model, which assumes that cholinergic projections are part of the diffuse modulatory systems^10,12^ globally affecting cortical regions.

The hierarchical cluster analysis is an unsupervised multivariate statistical method to analyze the internal relationships between data elements. It has been extensively applied, among other clustering methods, to measure connectivity in fMRI resting-state data^32^; analyze fMRI signals to infer functional connectivity^33^; extracellular electrophysiological properties of rat ventral tegmental area neurons^34^; or distribution of tau and amyloid deposits in the cerebral cortex^35^. Zilles’ group used quantitative multiple-receptor expression to reveal the hierarchical organization of neocortical areas in rats^36^, in the human cingulate cortex^37^, in Broca region^38^ and in the inferior parietal lobule^39^. An attempt was made in mice, using subjectively weighted strength of connections in anatomic modules to infer cortical organization^40^. Using different Cre driver lines to selectively label unique excitatory cell populations and quantitatively analyze axon terminal densities, Harris et al.,^41^ identified six cortical modules in mice: prefrontal, lateral, medial, somatomotor, visual, and auditory. Three of these overlap with the clusters we identified based on the cortical projection patterns of BFC neurons. Interestingly, the six-module cortical organization remained after adding thalamocortical input from 29 thalamic nuclei.

We remark that no preconceived hierarchical model was applied to the selection of cortical injection sites during the design of the experiment. The 30 cortical areas were selected purely based on the ontology, without considering their potential associations with the BFC system. We applied two additional tests to demonstrate that the observed hierarchical organization is not an artifact generated by the method. Firstly, we computed the significance of the branches of the dendrogram by using a bootstrap method, which confirmed that the 3 primary subnetworks are segregated at above 90% confidence (Extended Data Fig. 4). Secondly, we computed the principal component analysis from the correlation matrix, which revealed the same 3 clusters, including the same cortical areas as identified by the hierarchical clusters, with the three largest eigenvalues (Fig. 5).

### Hierarchical clustering: Technical considerations

Single-cell electrophysiology, quantitative anatomical tracing, and single-cell RNAseq furnished ample evidence suggesting that cortico-cortical connections in the mammalian brains are hierarchically organized^41–43^. Since hierarchy can be investigated at different scales, various morphological or physiological features can be measured. For anatomical features, connection strengths, and distance, markers for myelo-, cyto-, receptor architecture, cell density, etc., can be quantified. As we elaborate below, there are different methods for hierarchical clustering and for creating the dendrograms. It is unclear how the various anatomical and functional factors contribute to the hierarchy. We calculated cell density from spherical volumes of 300 μm radius centered on the cell body. This 300 μm radius was selected based on the probability of connections with other neurons and the dendritic and axonal arborization pattern of cholinergic neurons^16,21,44^. Although analysis with radius values from 100 μm to 500 μm did not change the general shape of the spatial density correlation matrix of Fig. 3, using a 300 μm radius articulated the three large networks the best. Since the normalization procedure removed the overlap of injection sites within ontology-defined cortical areas (see METHODS), overlapping injection sites did not bias the number of cases in the construction of the correlation matrix. On the other hand, removing whole ontology categories could have changed the shape of the dendrogram^37^. In our analysis, removing the ‘unidentified’ M1 and S1 whisker cases (injections that were targeted only using atlas coordinates without electrophysiology) indeed made a difference in the matrix and the dendrogram: including the ‘unidentified’ categories made the somatic sensory-motor network more compact, while removing them resulted in splitting this network in two subnetworks.

The dendrogram is a summary of the distance matrix, and, as such, information is lost. The consequence of this information loss is that the dendrograms are most accurate close to the leaves, showing which items are very similar. If the dendrogram is viewed separately from the correlation matrix, the location of the clades can be on either side of another clade within the same network. Clades (clusters) close to each other in the reordered correlation matrix, like S1-whisker/amygdala in Fig. 3, may belong to different major networks because they show increased similarity to clades in other networks. The median cell distance between each pair of cortical projection populations correlates with their respective Euclidean row vector distance in the correlation matrix (Extended Data Fig. 5. with Pearson’s *r* = 0.6292; *p* < 0.01). Since cortico-cortical connections show a significant correlation with injection distance^16^, one can assume that the similarity matrix of Fig. 3 relates to cortical connection strength.

While each cluster can be visualized in 3D and is in a specific space in the brain (Fig. 7), the shape of the dendrogram is a poor indicator of the number of clusters that coexist at each level, therefore we numbered them for easy explanation. Since the clusters are arranged in a hierarchy, to associate these clusters with cortical functional group compositions, one needs to draw cut lines parallel with the long axis of the dendrogram; observations that are joined together above the lines are in clusters. In Extended Data Fig. 6, we included 3 scenarios generating three, six and eighteen clusters and displayed them with the corresponding Silhouette plots^45^ to explain the composition of clusters. Accordingly, one can envisage combinations along the hierarchy of how the original 3 large clusters can be broken up into smaller and smaller clusters, eventually into many single clusters, segregated along functional cortical targets. These clusters may represent the flow of information.

### Anatomical connections and functional interactions within the three networks

There is extensive anatomical and functional interaction between M1FL and S1FL^46–49^; and sensorimotor integration between these body parts could be in part mediated by cholinergic *cluster 4*. S1 also projects to S2^47^ and *cluster 13* may modulate the interaction between S1HL and S2. Furthermore, S1 orofacial area, M1FL, S1FL are heavily interconnected^50^ and *clusters 10* and *16* could jointly innervate these body parts. Corroborating that visual, auditory, and entorhinal areas show only very sparse projection to M1 areas, we do not have cholinergic clusters that would jointly innervate these areas. Similarly, there is a lack of spatial density correlation between these areas in Fig. 3. Despite the RS region projections to M1^51–53^, M1 and RS show negative spatial density correlation (Fig. 3) and no joint cholinergic clusters innervating these regions either. The discrepancy between anatomical tracing and the spatial density correlation matrix may be related to the ‘fibers of passage’ problem, a common shortcoming in tracing experiments. Interestingly, whisker and forepaw regions in M1 receive different sets of corticocortical inputs^50^; correspondingly, M1-whisker, as a single-leafed clade, connects to the M1FL/S1FL clade. S1-whisker has direct projections to M1-whisker and S2-whisker^54^ and the cophenetic distance is 2.19, half of the maximum distance (Table S3).

Similarly, decades of research have found both anatomical and functional relationships among the identified auditory clusters (*clades 1,2,6*; Fig. 4). Most clearly, there are direct projections between A1 and A2 areas as well as shared thalamic inputs^55^. There are also reciprocal connections between the auditory thalamus and insular cortex^56,57^, and connectivity between both the auditory thalamus and auditory cortex to the amygdala^58^. Finally, there are auditory cortical projections to POR^59^ and the insular cortex is strongly interconnected with the amygdala^60^. In addition to the anatomical evidence that supports the relationship among these clusters, many studies have supported the functional connectivity between these regions, including lesions of the insular cortex that interfere with auditory processing^61^ and lesions of the POR resulting in impairments of auditory fear conditioning^62^. Arguably, the most extensively studied among these is the functional relationship between the amygdala and the auditory cortex, especially in relation to auditory learning, memory and auditory cortical plasticity, which is thought to be mediated via the BFCS^63,64^. In sum, rather than displaying a simple rostral-caudal relationship, these algorithmically identified clusters potentially reflect a functional network and provide a scaffolding for the cholinergic system to co-modulate these functionally and anatomically related regions.

Cortical visual processing is modulated by behavioral context; in V1 visual stimulation evoked responses are modulated by attention^65–68^, expectation^69,70^, reward^71,72^, locomotion^73,74^ and auditory processing^75^. In mice, direct cortico-cortical connections were proposed to play a role in the alteration of visual perception during specific behavioral tasks. Yet, the behavioral states during which the modulations occur overlap with brain states known to be regulated by cholinergic cortical neuromodulation such as arousal^76^, attention^77^, and information processing^78–82^. This suggests that cholinergic signaling interacts directly or indirectly (through the modulation of local inhibition) with the cellular mechanisms responsible for the integration of top-down feedback and bottom-up visual inputs. The cholinergic system facilitates activity propagation from V1 to the frontal cortex, and this process is linked to conscious detection of visual stimuli referred to as to global ignition^83^. In the correlation matrix of Fig. 2 and the rearranged matrix of Fig. 3, V1-auditory spatial density correlation coefficients are around 0.2, and the cophenetic distance on the dendrogram is 4.3; the maximum in our analysis that would suggest that these cortical areas are independently modulated by the cholinergic system. On the other hand, the 2.7 Euclidian distance is much less than the largest Euclidian distance (4.3; Table S3), suggesting that a cholinergic interaction between A1-V1 cannot be neglected.

Our earlier anatomical data^16^ and the current demonstration of the hierarchical organization of Ch projection strongly support the notion that the BFCS may provide a scaffolding for coordinated activity between remote yet associated cortical areas. It is hypothesized that contextual information provided by the hippocampus can trigger the recall of a past event during episodic memory through the activation of the mPFC neuronal ensembles^84^. This interaction may be, in part, mediated by the cholinergic system. Indeed, results showing concurrent coordinated ACh release in PFC and hippocampus^85^ during training on a rewarded working memory task, and this finding corroborates that the cholinergic branches to the hippocampus and the mPFC (PrL and IL) have a cophenetic distance of 1.8 with Euclidian distance between 1.72–1.83; (Table S3) are relatively close in the dendrogram (Extended Data Fig. 4).

### Clustered organization: A functional interpretation

As discussed in a symposium review^86^, *in vivo* electrophysiological data^87^, including experiments from our laboratory^88^, suggest that the forebrain cholinergic system is capable of behavior-dependent modulation of cortico-cortical functional connectivity, enabling information exchange between interconnected cortical regions.

It was noted already in a review more than 30 years ago^89^, that certain afferents to BFC neurons contact widespread portions of the system while other inputs target relatively restricted portions of the BFCS. Generalized behavioral functions such as sleep/wake cycles may be mediated in part through relatively diffuse inputs, such as noradrenergic afferents from the locus coeruleus^90^. Indeed, the structural connectivity between the noradrenergic and cholinergic systems affect network dynamics required to support adaptive behavior based on human imaging data^91^. Restricted afferents, including peptidergic and other forebrain inputs, may be related to more specific cortical processes^92^. In a recent study, we identified inputs to cholinergic cell groups projecting to M1/M2, OFC, mPFC^17^, entorhinal cortex, and the hippocampus (Gielow et al., 2018, SFN Abstract). Accordingly, the BFC neurons targeting each of these cortical areas receives a specific combination of afferents.

It is possible that the momentary combination of active inputs could select specific Ch nodes according to the hierarchical blueprint (Figs. 3, Extended Data Fig. 4). This organization may support a mechanism by which behavior-dependent modulation of specific cognitive processes is aligned with specific input-output wiring patterns of designated cholinergic cell groups. It is unclear how (i) specific inputs to Ch cells, (ii) local collaterals among Ch cell neurons in the BF, (iii) cortical axonal arborization of Ch cells in addition to (iv) secondary non-cholinergic connections participate in this mechanism. According to ours and an earlier study^93^ using well-localized small retrograde tracer injections into homologue areas of S1, S2, and M1, only 1–2% of the projection neurons were double labeled. While it is obscure how cholinergic clusters participate in the coordination of cognitive processes, our paper provides a unified conceptual framework of a hierarchically organized network of nodes to furnish testing of various functional hypotheses.

Recent studies using ACh indicators and large-scale imaging in mice demonstrate^94^, in contrast to traditional models implying a global influence of ACh throughout the cortex^95^, that various behavior states are associated with distinct spatiotemporal patterns of ACh release in the cortex. Moreover, cholinergic signaling is independent across different cortical areas^94^, consistent with the hierarchical, segregated network model presented in this paper. Another study in rats using DREADD technology combined with *in vivo* resting-state fMRI^96^ showed that selective activation of the cholinergic neurons in the BF, resulted in a reduction of resting-state neural activity in the default network, including the cingulate (Cg) and retrosplenial (RS) cortex, but did not change activity in the task-positive networks represented by seed regions in the S1, S2, M1, M2 and insular cortex. Again, these results support our data that cholinergic branches to the Cg/RS cortex are separated from the cholinergic clusters to the SSM and insular cortex, the latter is included in the Auditory network.

### Online content

Any methods, additional references, Nature Portfolio reporting summaries, source data, extended data, supplementary information, acknowledgements, peer review information; details of author contributions and competing interests; and statements of data and code availability are available at…

## Methods

### Animals

Animals were treated in accordance with the National Research Council’s “Guide for the Care and Use of Laboratory Animals.” Experiments were performed with the approval of the Institutional Animal Care and Use Committee of Rutgers University and by Penn State University College of Medicine. Data from Sprague-Dawley and ChAT::Cre rats^97^ were used as described in the original papers^9,16,17,24^. Rats were housed in cages of one to three animals on a 12-hr-light/12-hr-dark cycle at Rutgers University. Another group of Sprague-Dawley rats were housed at the Department of Neuroscience and Anatomy at Penn State University College of Medicine in Hershey, Pennsylvania under a collaborative agreement with Prof. Kevin Alloway. Code of the animals, treatment procedures, data related to injection sites, etc. are summarized in Table S1

### Surgeries, tracer injections (Fast Blue [FB] Fluoro-Gold [FG] or virus injections)

Each animal was anesthetized with an intramuscular dose of ketamine (20 mg/kg) and xylazine (6 mg/kg) or with isoflurane (1–4%) inhalation in O_2_. Its head was immobilized in a standard stereotaxic frame, and it received atropine methyl nitrate (0.05 mg/kg, intramuscular), chloramphenicol (50 mg/kg, intramuscular), and dexamethasone (0.5 mg/kg, intramuscular) to aid in ventilation and to help prevent subsequent infection and inflammation.

Electrophysiological recording and stimulation were used to select appropriate tracer injection sites in the somatosensory, motor, and auditory cortices. For somatosensory cortex injections, specific vibrissae regions were stimulated with fine sticks as a recording electrode was lowered into the cortex to localize sensory activity. The somatotopic representation in the motor cortex was determined by using low intensity intracortical microstimulation and was administered to evoke brief twitches of muscles controlling the limbs or whiskers as described^25^. The auditory cortex was identified with tungsten micro electrodes lowered to layers IV-V of auditory cortex. A1 was defined by a general progression of low to high characteristic frequency (CF) along the posterior to anterior axis^9^. Anterior placements that resulted in a reversal in the CF progression were defined as lying in the anterior auditory field (AAF^28^). The posterior dorsal auditory field (PDAF^28^) belt area was identified as more responsive to noise than to tone. The suprarhinal auditory field (SRAF^29,30^), was identified as the auditory responsive region just above the rhinal sulcus.

Following the electrophysiological identification of selected representations in the somatosensory, motor, and auditory cortices, Fast Blue (FB; Polysciences, Warrington, PA) was deposited by pressure using glass pipettes (tip diameter, 40–80 μm) injections and Fluoro-Gold (FG; Fluorochrome, Denver, CO) by iontophoresis (negative, pulsed DC current, 7 μA; 7-second on–off cycles; 20 minutes), both 2% solution in purified water. FB and FG injections in the prefrontal^16^, insular^16^, perirhinal^24^, postrhinal^24^, entorhinal cortex^24^ were done only using topographic coordinates of the Paxinos/Watson^100^ atlas without electrophysiological identification and has been described earlier. Additional FB or FG injections were deposited in the M2, S2 and retrosplenial cortex using atlas coordinates. Visual cortical sites were located via intracortical local field recordings time-locked to stimulus presentation. Visual cortical responsiveness was queried using a 3M NaCl micropipette during presentation of strobe light stimulation of the contralateral visual field (Gielow et al., 2013, SFN Abstract). Visually responsive sites received iontophoretic injection of FB.

After a survival period of 6–10 days, the animals were deeply anesthetized with isoflurane supplemented with urethane (1.5 ml of a 0.35 g/ml solution) and transcardially perfused with 0.9% saline, followed by 4% paraformaldehyde in 0.1 M phosphate buffer (PB). The brains were removed from the skull, postfixed overnight in the same fixative, and stored in 0.1 M phosphate buffer with 30% sucrose. Similar procedures were followed for anesthesia, postoperative care, and perfusion in both Universities. Brains with somatic sensory and motor injections (n=35) were shipped from Penn State University College of Medicine to Rutgers University in PB with 30% sucrose between 2001–2010.

#### Virus injections.

Rabies monosynaptic virus tracing^98^ was used in Chat::Cre transgenic rats expressing Cre under the choline acetyl transferase promoter^98^. Rats were anesthetized with isoflurane (Isothesia, Henry Schein), and helper viruses^99^ (AAV-EF1a-FLEXTVA-mCherry and AAV-CA-FLEX-RG, UNC vector core) were first injected in the BF area that is enriched in auditory cortically projecting BF cholinergic cells^9^. In rats that were designed to study cholinergic projections to mPFC, orbitofrontal, motor cortex and the amygdala^17^, the helper viruses were injected via micropipette across five locations filling the right BF. The helper virus leads to the expression of mCherry fluorescent marker, TVA viral receptor, and rabies virus envelope glycoprotein (RG) in a Cre-dependent manner in cholinergic cells. Following 21 days of recovery, rats were again anesthetized (see above), and the specific cortical areas were injected with modified rabies pseudotyped with the avian virus envelope (EnvA G-deleted rabies e-GFP^99^ Salk Vector Core). Prior to virus injections, the auditory cortex was electrophysiologically identified as described above. In all other cases, cortical targets were identified only using topographic coordinates of the Paxinos/Watson atlas^100^. We limited the size of the rabies injection to prevent any spillover into adjacent cortical regions. In each case, a 0.2 μL injection was made in each identified cortical region. The modified rabies restricts infection to TVA receptor-expressing cells. The rabies virus lacks the RG necessary for transynaptic spread of the virus. This procedure results in specific infection of mCherry/TVA-expressing cholinergic cells (previously infected with helper viruses) and restricts transynaptic spread of the rabies virus to cells monosynaptically connected to these cholinergic neurons^101^. Double-labeled e-GFP (modified rabies) and mCherry (helper virus) cells are cholinergic neurons (starter cells) that projected to the target cortical region, whereas e-GFP-positive singly labeled cells indicate monosynaptic (afferent) input to the starter cells as described^9,17.^

### Tissue preparation, immunofluorescence, plotting labelled cells, Nissl staining and delineation of BF areas

50 μm-thick coronal sections were cut using a sliding microtome. Sections were divided into four series, so that a brain may be represented by every fourth section, with a 200 μm distance between adjacent sections. Two of the four series were processed for immunocytochemically identified cholinergic neurons, using fluorescently-tagged antibodies against choline-acetyltransferase (ChAT). To confirm specificity of viral targeting, sections from 2 cases of helper virus-injected animal were immunostained for ChAT. For details on tissue processing, see the original papers. A computer-microscopy system equipped with the Neurolucida^R^ software (v.7.50.4 MBF Bioscience, MicroBrightField, Inc.) was used to trace all sections and significant contours (i.e. Corpus Callosum, Lateral Ventricle, Lateral Olfactory Tract, Optic Chiasm, Anterior Commisure, Internal Capsule, Third Ventricle, Dorsal Third Ventricle, Hippocampus, Fornix, Stria Terminalis, Stria Medullaris, Caudate Nucleus and Zona Incerta), and an epifluorescence microscope was used to visualize and map all retrogradely labeled cells for FB, FG and ChAT present in the BF. After plotting, the coverslips were removed, and the same slides were prepared with a thionin Nissl stain. Nissl-stained sections were illuminated with bright-field and aligned to existing images or to existing Neurolucida maps previously created under fluorescence. Nissl-images of the sections with cholinergic projection neurons were scanned and warped to the template brain (see Anatomical Data Integration Pipeline). BF cytoarchitectonic boundaries were drawn on the template brain sections using the QGIS program for comparing the number of neurons in specific BF regions projecting to specific ontologically identified cortical regions.

### Database, annotation, software

We used PostGIS plugin framework around Postgres database (RDBMS: relational database management system) to store image data and corresponding vectorial data, including cell locations and structure delineations. This solution provides us with a complete set of functions for analyzing geometric components, determining spatial relationships, and manipulating delineations. For 2D data visualization we used the QGIS Mapping Client. This framework provides us with very fast and smooth visualization of high-resolution images as well as vectorial data.

### Image and vectorial data registration

We created an image registration tool (Java) for anatomic image and vectorial data registration. We extended an ImageJ plugin to be suitable for this purpose^102^. This tool can use delineations as well as image data to guide the registration and can communicate directly with the Postgres database. The tool is using the combination of affine and BSpline-based elastic transformation for registration. The registration process starts with section matching. Each section of the experimental brain is visually matched with the corresponding section in the reference brain. Since there were differences in terms of cutting angle between experiments, we created a special tool to aid section matching. This tool (Java/ImageJ) can generate high resolution virtual sections of the reference brain based on user inputs by cutting it in different angles real time and provide the high-resolution image that matches with the experimental brain section image. The used cutting angle has been considered during the final computation of cell positions in the reference brain. After section matching, we visually inspect identical reference points on both the experimental and reference section. Based on those reference points we apply the registration transformation. We registered 106 cortical injections in 73 brains into a single reference brain using the registration tool described above. Fluorescent-labeled cholinergic and non-cholinergic neurons were mapped in the BF using the Neurolucida^R^ (MBF Bioscience) data-acquisition system. After mapping, coverslips were removed and sections were Nissl-stained, realigned with the corresponding maps, and scanned using NL system. To validate the registration process of experimental sections we compared the registered cytoarchitectonic zones to their correspondent zone on the reference section they registered into. On randomly selected sections we used the Dice coefficient^103^ to measure the difference,

(1) $$D=\frac{2\;\text{A}(\text{X}\cap Y)}{A(X)+A(Y)}$$

where X, Y are arbitrary geometries and A is the area function (Extended Data Fig. 3).

### Injection sites in Paxinos-coronal-space

First, we adjusted the images of the Paxinos-atlas with the corresponding outline and registered the injections sites from the Nissl-images of the brain sections using elastic transformation. We measured the participation of injection site I in cortical region R by dividing the volume of their intersection by the volume of I (formula 2). Additionally, we expressed how much injection site’A’is covered by injection site ‘B’ by measuring the volume of intersection of injection site A with injection site B and divide it by the volume of injection site A (formula 3).


(2) $${\text{P}}_{\text{I}}(\text{R})=\frac{V(I\cap R)}{V(I)}$$

(3) $${\text{O}}_{\text{A}}(\text{B})=\frac{V(A\cap B)}{V(A)}$$

### Construction of spatial correlation matrices

Projection cholinergic neurons from all experimental brains were registered to the reference brain. We sampled the BF cholinergic space at multiple points to determine the composition of cortical projection neurons according to their cortical targets. The sampling points were the projection neuron positions in the BF. We defined a sphere of 300 μm radius around each projection neuron (n =5,674) and counted the number of neighbors projecting to any cortical injection sites. The injection sites were grouped according to 30 ontological categories (Table S2). Thus, for each sampling point, we made 30 measurements at its possible target projections, which provided a target-projection-vector (TPV) of 30 integer values. Then, we combined these vectors into a matrix where each row represented a TPV with the number of cortical targets shared within the proximity of the sampling location. When counted the projection neurons around each cell, we also had to account for overlapping injection sites. We applied a normalization to compensate for the multiple labeling of the same projection area. At each measurement point of the cholinergic cell space, we checked whether any neighboring cell projected to cortical targets with overlapping injection sites and computed a normalization constant (Eq. 4) to be applied to the TPV. This normalization constant (*C*_j_) was defined for a given injection site *I*_j_ covering cortical region R as:

(4) $$C_{j}=\frac{\text{v}\left(I_{j}\right)}{\sum_{I_{k}\in {\text{I}}_{R}\;\text{V}\left(I_{j}\cap I_{k}\right)}},$$

where I_*R*_ is the set of injections covering cortical region *R,* and V is the volume of injections

At any given sampling location **p**, we calculated the normalized number of projection neurons of projection population **j**

(5) $${\tilde{N}}_{p,j}=C_{j}\times N_{p,j}$$

where N _P_,_j_ is the original number of projection neurons from the cholinergic population **j**, and C_j_ is the normalization constant (Formula 4).

After normalization, the dataset was ready to be analyzed for the composition similarity of BFC ensembles regarding their cortical projection patterns To do that, we computed Pearson’s correlations (formula 6) between all pairs of column vectors (30 × 5,674) except the column vector itself, resulting in a 30 × 30 matrix of Figure 3 representing the most likely composition of neurons within 300 μm ensembles of neurons identified by their cortical projections.


(6) $$r=\frac{\sum \left(x_{i}-\bar{x}\right)\left(y_{i}-\bar{y}\right)}{\sqrt{\sum {\left(x_{i}-\bar{x}\right)}^{2}\sum {\left(y_{i}-\bar{y}\right)}^{2}}}$$

For visualization purposes, we rendered negative, zero and positive correlations with the shades of blue, white and red, respectively. Red areas represent pairs of cortical areas sharing similar composition of cholinergic neurons projecting to them and other cortical areas from local ensemble of BFC neurons. Next, we applied hierarchical clustering^104^ to the correlation matrix using Euclidian distance and *MAX (complete) linkage* using the open-source R statistical framework (Figure 3). The program first generates the distance matrix between the rows. Each row is handled as a single cluster. The algorithm is iterative and at each iteration it tries to find the closest pair of clusters - according to MAX linkage - and merge them into one. At each iteration point the program reorganizes the matrix rows so that the in between cluster will be minimal. As a result, the final matrix was organized to reflect the compositional similarity of BFC neurons in terms of the similarity of their projection patterns to cortical targets.

### Statistical analysis of cluster dendrograms

Hierarchical clustering generates dendrograms no matter how segregated and hierarchically organized the dataset is. To assert the level of uncertainty associated with the clusters we used *pvclust* in R package^27^ that implemented multiscale bootstrap resampling. This method associates a p value (0<p<1) with each branch based on estimating how unlikely is to obtain a given cluster configuration by-chance after random resampling of the dataset. In Extended Data Fig. 4 is the P-values extended dendrogram from Figure 3. The red numbers at branching points represent Approximate Unbiased p values^26^. We used cophenetic correlation^104^ to measure how faithfully the dendrogram preserves the pairwise Euclidean distances between row vectors of correlation coefficients for each cortical area. Cophenetic distance between two leaves on a dendrogram is the intergroup dissimilarity at which the two are first combined into a single cluster. We used ‘complete linkage’ in our hierarchical clustering method, which is the distance between the outermost data points between two clusters. The cophenetic correlation coe cient (r_c_=0.88) is the result of the Pearson correlation between the cophenetic distance and the Euclidean distance for each row vector pair.

Furthermore, to partition the dendrogram we used the *Silhouette* method^45^, which finds the optimal cut of the dendrogram (cluster number) that represent the most coherent cluster separation in our data. The silhouette value shows how similar an object is to its own cluster compared to the other clusters. The silhouette score^45^ ranges from −1 to +1. A high value indicates that the object well matches the cluster where it belongs and poorly matches the other clusters. For the Silhouette, we compute a score by measuring the object’s average distance (Euclidean) from objects of the other clusters and iterating that for each cluster, including its own. Then, we compare the average of its cluster to the neighboring clusters’ averages. We find the neighboring cluster with the smallest difference and compute the Silhouette score as the ratio of this difference to the absolute average distance of this neighbor. We obtained the highest silhouette score (s=0.55) when we cut the dendrogram into three parts, hence confirming the 3 main clusters also apparent by visual observation (Extended Data Fig. 6).

### Visualization methods

The aim is to find high-density spaces for single target populations. Using TPV (see above), we can assess the projection neuron densities at any sampling point in the cholinergic space. We computed the average neuron density for each target across all sampling points. Next, we visualize all the sampling points where the density of a specific target is above its average (Fig. 4B).

### Nomenclature of cortical and Basal Forebrain areas (Ontology hierarchies)

Table S2 lists in *column* A cortical regions based on cytoarchitectonic delineations. Columns B-G lists categories where a specific region is encompassed in larger and larger volumes. We used in our work mostly the terms and abbreviations defined in *column* B. While we tried to adhere as much as possible to terms used in the fifth edition of the Paxinos-Watson atlas^100^, we applied a few updated terms based on our studies using electrophysiology in the auditory, somatic sensory and motor cortical areas. For example, body parts in M1 and S1 cortex were specified using intraoperative electrophysiology. Auditory cortical areas were designated as A1, AAF, PDAF, SRAF as defined electrophysiologically as explained under “FB and FG tracer injections in cortical areas”. Table S1 in column G explains the involvement of cortical areas according to the Paxinos-Watson atlas^100^ that was used for registering injection sites. For example, injection case 13082FG was designated as AAF cortex, this injection encompasses parts of the secondary dorsal auditory area, barrel field, S1 upper lip and S2 regions as delineated in the Paxinos-Watson atlas (compare Figure S1 and Table S1). Perirhinal (PER, areas 35 and 36) and postrhinal (POR) areas were defined as Burwell and Amaral^106^. MEC and LEC for the medial and lateral entorhinal areas are used as Witter et al.^107^.

## Data Availability

All datasets in the present paper will be publicly available upon acceptance of this paper at http://zaborszkylab.org/3DCholinergicSpace/.

## Abbreviations

a: caudal continuation of HDB
A1: primary auditory cortex
A2: secondary auditory cortex
AAF: anterior auditory field
Ach: acetylcholine
AL: nucleus of the ansa lenticularis
BF: basal forebrain
BFC: basal forebrain cholinergic
BFCS: basal forebrain cholinergic system
Cg1: cingulate cortex
Ch: cholinergic
ChAT: choline acetyltransferase
AIP: anterior insular cortex, posterior part
EA: extended amygdala
FB: Fast Blue
FG: Fluoro-Gold
GP: globus pallidus
HDB: horizontal limb of the diagonal band
ic: internal capsule
IDIGI: Insular cortex, dysgranular, granular parts
IL: infralimbic cortex
LEC: lateral entorhinal cortex
LO: lateral orbital cortex
M1: primary motor cortex
M1FL: primary motor cortex, forelimb
M1W: primary motor cortex, whisker
M2: secondary motor cortex
MEC: medial entorhinal cortex
MO: medial orbital cortex
mPFC: medial prefrontal cortex
MS: medial septum
OFC: orbitofrontal cortex
PDAF: posterior dorsal auditory field
PFC: prefrontal cortex
PL: prelimbic cortex
POR: postrhinal cortex
Prh: perirhinal cortex
PrL: prelimbic cortex
RS: retrosplenial cortex
S1: primary somatosensory cortex
S1FL: primary somatosensory cortex, forelimb
S1HL: primary somatosensort cortex, hindlimb
S1W: primary somatosensort cortex, whisker
S2: secondary somatosensory cortex
SDC: spatial density correlation
SPL: sound pressure level
SRAF: suprarhinal auditory field
SSM: somatic sensory-motor
SIB: substantia innominata, basal part
TPV: target-projection vector
V1M: primary visual cortex, monocular area
V2ML: secondary visual cortex, mediolateral area
VDB: vertical limb of the diagonal band
VO: ventral orbital cortex
VP: ventral pallidum
